# A survey on platforms for big data analytics

**DOI:** 10.1186/s40537-014-0008-6

**Published:** 2014-10-09

**Authors:** Dilpreet Singh, Chandan K Reddy

**Affiliations:** Department of Computer Science, Wayne State University, Detroit, MI 48202 USA

**Keywords:** Big data, MapReduce, graphics processing units, scalability, big data analytics, big data platforms, k-means clustering, real-time processing

## Abstract

The primary purpose of this paper is to provide an in-depth analysis of different platforms available for performing big data analytics. This paper surveys different hardware platforms available for big data analytics and assesses the advantages and drawbacks of each of these platforms based on various metrics such as scalability, data I/O rate, fault tolerance, real-time processing, data size supported and iterative task support. In addition to the hardware, a detailed description of the software frameworks used within each of these platforms is also discussed along with their strengths and drawbacks. Some of the critical characteristics described here can potentially aid the readers in making an informed decision about the right choice of platforms depending on their computational needs. Using a star ratings table, a rigorous qualitative comparison between different platforms is also discussed for each of the six characteristics that are critical for the algorithms of big data analytics. In order to provide more insights into the effectiveness of each of the platform in the context of big data analytics, specific implementation level details of the widely used k-means clustering algorithm on various platforms are also described in the form pseudocode.

## Introduction

This is an era of Big Data. Big Data is driving radical changes in traditional data analysis platforms. To perform any kind of analysis on such voluminous and complex data, scaling up the hardware platforms becomes imminent and choosing the right hardware/software platforms becomes a crucial decision if the user’s requirements are to be satisfied in a reasonable amount of time. Researchers have been working on building novel data analysis techniques for big data more than ever before which has led to the continuous development of many different algorithms and platforms.

There are several big data platforms available with different characteristics and choosing the right platform requires an in-depth knowledge about the capabilities of all these platforms [1]. Especially, the ability of the platform to adapt to increased data processing demands plays a critical role in deciding if it is appropriate to build the analytics based solutions on a particular platform. To this end, we will first provide a thorough understanding of all the popular big data platforms that are currently being used in practice and highlight the advantages and drawbacks of each of them.

Typically, when the user has to decide the right platforms to choose from, he/she will have to investigate what their application/algorithm needs are. One will come across a few fundamental issues in their mind before making the right decisions.

- How quickly do we need to get the results?
- How big is the data to be processed?
- Does the model building require several iterations or a single iteration?

Clearly, these concerns are application/algorithm dependent that one needs to address before analyzing the systems/platform-level requirements. At the systems level, one has to meticulously look into the following concerns:

- Will there be a need for more data processing capability in the future?
- Is the rate of data transfer critical for this application?
- Is there a need for handling hardware failures within the application?

In this paper, we will provide a more rigorous analysis of these concerns and provide a score for each of the big data platforms with respect to these issues.

While there are several works that partly describe some of the above mentioned concerns, to the best of our knowledge, there is no existing work that compares different platforms based on these essential components of big data analytics. Our work primarily aims at characterizing these concerns and focuses on comparing all the platforms based on these various optimal characteristics, thus providing some guidelines about the suitability of different platforms for various kinds of scenarios that arise while performing big data analytics in practice.

In order to provide a more comprehensive understanding of the different aspects of the big data problem and how they are being handled by these platforms, we will provide a case study on the implementation of k-means clustering algorithm on various big data platforms. The k-means clustering was chosen here not only because of its popularity, but also due to the various dimensions of complexity involved with the algorithm such as being iterative, compute-intensive, and having the ability to parallelize some of the computations. We will provide a detailed pseudocode of the implementation of the k-means clustering algorithm on different hardware and software platforms and provide an in-depth analysis and insights into the algorithmic details.

The major contributions of this paper are as follows:

- Illustrate the scaling of various big data analytics platforms and demonstrate the advantages and drawbacks of each of these platforms including the software frameworks.
- Provide a systematic evaluation of various big data platforms based on important characteristics that are pertinent to big data analytics in order to aid the users with a better understanding about the suitability of these platforms for different problem scenarios.
- Demonstrate a case study on the k-means clustering algorithm (a representative analytics procedure) and describe the implementation level details of its functioning on various big data platforms.

The remainder of the paper is organized as follows: the fundamental scaling concepts along with the advantages and drawbacks of horizontal and vertical scaling are explained in Section “Scaling”. Section “Horizontal scaling platforms” describes various horizontal scaling platforms including peer-to-peer networks, Hadoop and Spark. In section “Vertical scaling platforms”, various vertical platforms graphics processing units and high performance clusters are described. Section “Comparison of different platforms” provides thorough comparisons between different platforms based on several characteristics that are important in the context of big data analytics. Section “How to choose a platform for big data analytics?” discusses various details about choosing the right platform for a particular big data application. A case study on k-means clustering algorithm along with its implementation level details on each of the big data platform is described in Section “K-means clustering on different platforms”. Finally, the “Conclusion” section concludes our discussion along with future directions.

## Scaling

Scaling is the ability of the system to adapt to increased demands in terms of data processing. To support big data processing, different platforms incorporate scaling in different forms. From a broader perspective, the big data platforms can be categorized into the following two types of scaling:

- **Horizontal Scaling:** Horizontal scaling involves distributing the workload across many servers which may be even commodity machines. It is also known as “scale out”, where multiple independent machines are added together in order to improve the processing capability. Typically, multiple instances of the operating system are running on separate machines.
- **Vertical Scaling:** Vertical Scaling involves installing more processors, more memory and faster hardware, typically, within a single server. It is also known as “scale up” and it usually involves a single instance of an operating system.

Table 1 compares the advantages and drawbacks of horizontal and vertical scaling. While scaling up vertically can make the management and installation straight-forward, it limits the scaling ability of a platform since it will require substantial financial investment. To handle future workloads, one always will have to add hardware which is more powerful than the current requirements due to limited space and the number of expansion slots available in a single machine. This forces the user to invest more than what is required for his current processing needs.

**Table 1:** **A comparison of advantages and drawbacks of horizontal and vertical scaling**

**Scaling**	**Advantages**	**Drawbacks**
**Horizontal scaling**	➔ Increases performance in small steps as needed	➔ Software has to handle all the data distribution and parallel processing complexities
	➔ Financial investment to upgrade is relatively less	➔ Limited number of software are available that can take advantage of horizontal scaling
	➔ Can scale out the system as much as needed	
**Vertical scaling**	➔ Most of the software can easily take advantage of vertical scaling	➔ Requires substantial financial investment
	➔ Easy to manage and install hardware within a single machine	➔ System has to be more powerful to handle future workloads and initially the additional performance in not fully utilized
		➔ It is not possible to scale up vertically after a certain limit

On the other hand, horizontal scale out gives users the ability to increase the performance in small increments which lowers the financial investment. Also, there is no limit on the amount of scaling that can done and one can horizontally scale out the system as much as needed. In spite of these advantages, the main drawback is the limited availability of software frameworks that can effectively utilize horizontal scaling.

## Horizontal scaling platforms

Some of the prominent horizontal scale out platforms include peer-to-peer networks and Apache Hadoop. Recently, researchers have also been working on developing the next generation of horizontal scale out tools such as Spark [2] to overcome the limitations of other platforms. We will now discuss each of these platforms in more detail in this section.

### Peer-to-peer networks

Peer-to-Peer networks [3,4] involve millions of machines connected in a network. It is a decentralized and distributed network architecture where the nodes in the networks (known as peers) serve as well as consume resources. It is one of the oldest distributed computing platforms in existence. Typically, Message Passing Interface (MPI) is the communication scheme used in such a setup to communicate and exchange the data between peers. Each node can store the data instances and the scale out is practically unlimited (can be millions of nodes).

The major bottleneck in such a setup arises in the communication between different nodes. Broadcasting messages in a peer-to-peer network is cheaper but the aggregation of data/results is much more expensive. In addition, the messages are sent over the network in the form of a spanning tree with an arbitrary node as the root where the broadcasting is initiated.

MPI, which is the standard software communication paradigm used in this network, has been in use for several years and is well-established and thoroughly debugged. One of the main features of MPI includes the state preserving process i.e., processes can live as long as the system runs and there is no need to read the same data again and again as in the case of other frameworks such as MapReduce (explained in section “Apache hadoop”). All the parameters can be preserved locally. Hence, unlike MapReduce, MPI is well suited for iterative processing [5]. Another feature of MPI is the hierarchical master/slave paradigm. When MPI is deployed in the master–slave model, the slave machine can become the master for other processes. This can be extremely useful for dynamic resource allocation where the slaves have large amounts of data to process.

MPI is available for many programming languages. It includes methods to send and receive messages and data. Some other methods available with MPI are ‘Broadcast’, which is used to broadcast the data or messages over all the nodes and ‘Barrier’, which is another method that can put a barrier and allows all the processes to synchronize and reach up to a certain point before proceeding further.

Although MPI appears to be perfect for developing algorithms for big data analytics, it has some major drawbacks. One of the primary drawbacks is the fault intolerance since MPI has no mechanism to handle faults. When used on top of peer-to-peer networks, which is a completely unreliable hardware, a single node failure can cause the entire system to shut down. Users have to implement some kind of fault tolerance mechanism within the program to avoid such unfortunate situations. With other frameworks such as Hadoop (that are robust to fault tolerance) becoming widely popular, MPI is not being widely used anymore.

### Apache hadoop

**Figure 1:**
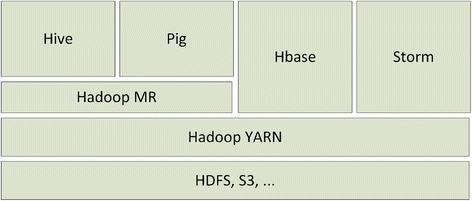
**Hadoop Stack showing different components.**

Apache Hadoop [6] is an open source framework for storing and processing large datasets using clusters of commodity hardware. Hadoop is designed to scale up to hundreds and even thousands of nodes and is also highly fault tolerant. The various components of a Hadoop Stack are shown in Figure 1. The Hadoop platform contains the following two important components:

- Distributed File System (HDFS) [7] is a distributed file system that is used to store data across cluster of commodity machines while providing high availability and fault tolerance.
- Hadoop YARN [8] is a resource management layer and schedules the jobs across the cluster.

#### MapReduce

The programming model used in Hadoop is MapReduce [9] which was proposed by Dean and Ghemawat at Google. MapReduce is the basic data processing scheme used in Hadoop which includes breaking the entire task into two parts, known as mappers and reducers. At a high-level, mappers read the data from HDFS, process it and generate some intermediate results to the reducers. Reducers are used to aggregate the intermediate results to generate the final output which is again written to HDFS. A typical Hadoop job involves running several mappers and reducers across different nodes in the cluster. A good survey about MapReduce for parallel data processing is available in [10].

#### MapReduce wrappers

A certain set of wrappers are currently being developed for MapReduce. These wrappers can provide a better control over the MapReduce code and aid in the source code development. The following wrappers are being widely used in combination with MapReduce.

- **Apache Pig** is a SQL-like environment developed at Yahoo [11] is being used by many organizations like Yahoo, Twitter, AOL, LinkedIn etc. **Hive** is another MapReduce wrapper developed by Facebook [12]. These two wrappers provide a better environment and make the code development simpler since the programmers do not have to deal with the complexities of MapReduce coding.
- Programming environments such as **DryadLINQ**, on the other hand, provide the end users with more flexibility over the MapReduce by allowing the users to have more control over the coding. It is a C# like environment developed at Microsoft Research [13]. It uses LINQ (a parallel language) and a cluster execution environment called Dryad. The advantages include better debugging and development using Visual Studio as the tool and interoperation with other languages such as standard .NET.

In addition to these wrappers, some researchers have also developed scalable machine learning libraries such as Mahout [14] using MapReduce paradigm.

#### Limitations of MapReduce

One of the major drawbacks of MapReduce is its inefficiency in running iterative algorithms. MapReduce is not designed for iterative processes. Mappers read the same data again and again from the disk. Hence, after each iteration, the results have to be written to the disk to pass them onto the next iteration. This makes disk access a major bottleneck which significantly degrades the performance. For each iteration, a new mapper and reducer have to be initialized. Sometimes the MapReduce jobs are short-lived in which case the overhead of initialization of that task becomes a significant overhead to the task itself. Some workarounds such as forward scheduling (setting up the next MapReduce job before the previous one finishes) have been proposed. However, these approaches introduce additional levels of complexity in the source code. One such work called HaLoop [15] extends MapReduce with programming support for iterative algorithms and improves efficiency by adding caching mechanisms. CGL MapReduce [16,17] is another work that focuses on improving the performance of MapReduce iterative tasks. Other examples of iterative MapReduce include Twister [18] and imapreduce [19].

### Spark: next generation data analysis paradigm

Spark is a next generation paradigm for big data processing developed by researchers at the University of California at Berkeley. It is an alternative to Hadoop which is designed to overcome the disk I/O limitations and improve the performance of earlier systems. The major feature of Spark that makes it unique is its ability to perform in-memory computations. It allows the data to be cached in memory, thus eliminating the Hadoop’s disk overhead limitation for iterative tasks. Spark is a general engine for large-scale data processing that supports Java, Scala and Python and for certain tasks it is tested to be up to 100× faster than Hadoop MapReduce when the data can fit in the memory, and up to 10× faster when data resides on the disk. It can run on Hadoop Yarn manager and can read data from HDFS. This makes it extremely versatile to run on different systems.

#### Berkeley data analytics stack (BDAS)

**Figure 2:**
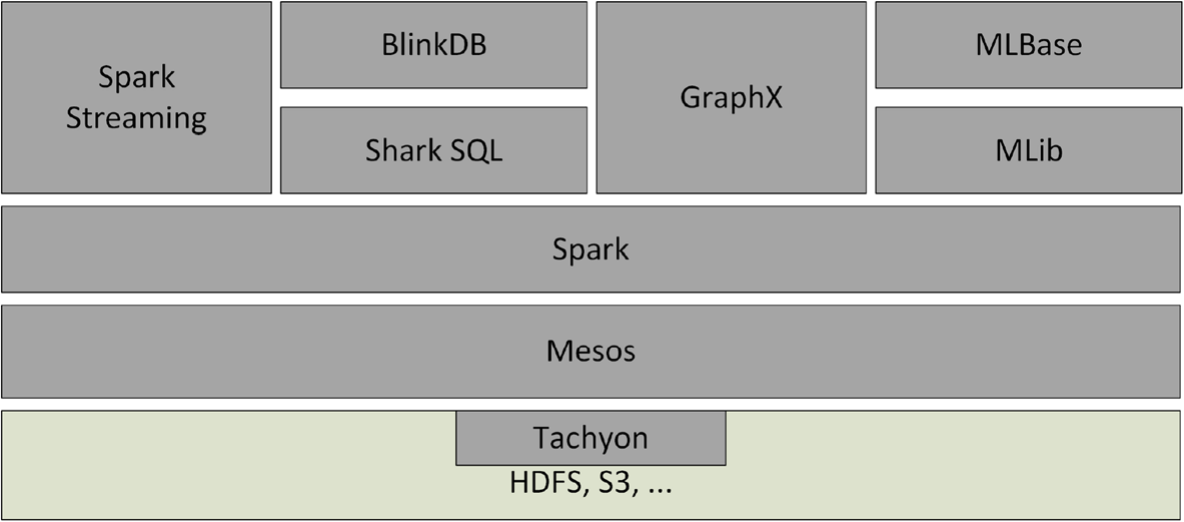
**An illustration of Berkeley Data Analysis Stack and its various components [** 20 **].**

The Spark developers have also proposed an entire data processing stack called Berkeley Data Analytics Stack (BDAS) [20] which is shown in Figure 2. At the lowest level of this stack, there is a component called Tachyon [21] which is based on HDFS. It is a fault tolerant distributed file system which enables file sharing at memory-speed (data I/O speed comparable to system memory) across a cluster. It works with cluster frameworks such as Spark and MapReduce. The major advantage of Tachyon over Hadoop HDFS is its high performance which is achieved by using memory more aggressively. Tachyon can detect the frequently read files and cache them in memory thus minimizing the disk access by different jobs/queries. This enables the cached files to be read at memory speed. Another feature of Tachyon is its compatibility with Hadoop MapReduce. MapReduce programs can run over Tachyon without any modifications. The other advantage of using Tachyon is its support for raw tables. Tables with hundreds of columns can be loaded easily and the user can specify the frequently used columns to be loaded in memory for faster access.

The second component in BDAS, which is the layer above Tachyon, is called Apache Mesos. Mesos is a cluster manager that provides efficient resource isolation and sharing across distributed applications/frameworks. It supports Hadoop, Spark, Aurora [22], and other applications on a dynamically shared pool of resources. With Mesos, scalability can be increased to tens of thousands of nodes. APIs are available in java, python and C++ for developing new parallel applications. It also includes multi-resource scheduling capabilities.

The third component running on top of Mesos is Spark which takes the place of Hadoop MapReduce in the BDAS architecture. On the top of the stack are many Spark wrappers such as Spark Streaming (Large Scale real-time stream processing), Blink DB (queries with bounded errors and bounded response times on very large data) [23], GraphX (Resilient distributed Graph System on Spark) [24] and MLBase (distributed machine learning library based on Spark) [25].

Recently, BDAS and Spark have been receiving a lot of attention due to their performance gain over Hadoop. Now, it is even possible to run Spark on Amazon Elastic Map-Reduce [26]. Although BDAS consists of many useful components in the top layer (for various applications), many of them are still in the early stages of development and hence the support is rather limited. Due to the vast number of tools that are already available for Hadoop MapReduce, it is still the most widely used distributed data processing framework.

## Vertical scaling platforms

The most popular vertical scale up paradigms are High Performance Computing Clusters (HPC), Multicore processors, Graphics Processing Unit (GPU) and Field Programmable Gate Arrays (FPGA). We describe each of these platforms and their capabilities in the following sections.

### High performance computing (HPC) clusters

HPC clusters [27], also called as blades or supercomputers, are machines with thousands of cores. They can have a different variety of disk organization, cache, communication mechanism etc. depending upon the user requirement. These systems use well-built powerful hardware which is optimized for speed and throughput. Because of the top quality high-end hardware, fault tolerance in such systems is not problematic since hardware failures are extremely rare. The initial cost of deploying such a system can be very high because of the use of the high-end hardware. They are not as scalable as Hadoop or Spark clusters but they are still capable of processing terabytes of data. The cost of scaling up such a system is much higher compared to Hadoop or Spark clusters. The communication scheme used for such platforms is typically MPI. We already discussed about MPI in the peer-to-peer systems (see section “Peer-to-peer networks”). Since fault tolerance is not an important issue in this case, MPIs’ lack of fault tolerance mechanism does not come as a significant drawback here.

### Multicore CPU

Multicore refers to one machine having dozens of processing cores [28]. They usually have shared memory but only one disk. Over the past few years, CPUs have gained internal parallelism. More recently, the number of cores per chip and the number of operations that a core can perform has increased significantly. Newer breeds of motherboards allow multiple CPUs within a single machine thereby increasing the parallelism. Until the last few years, CPUs were mainly responsible for accelerating the algorithms for big data analytics.

**Figure 3:**
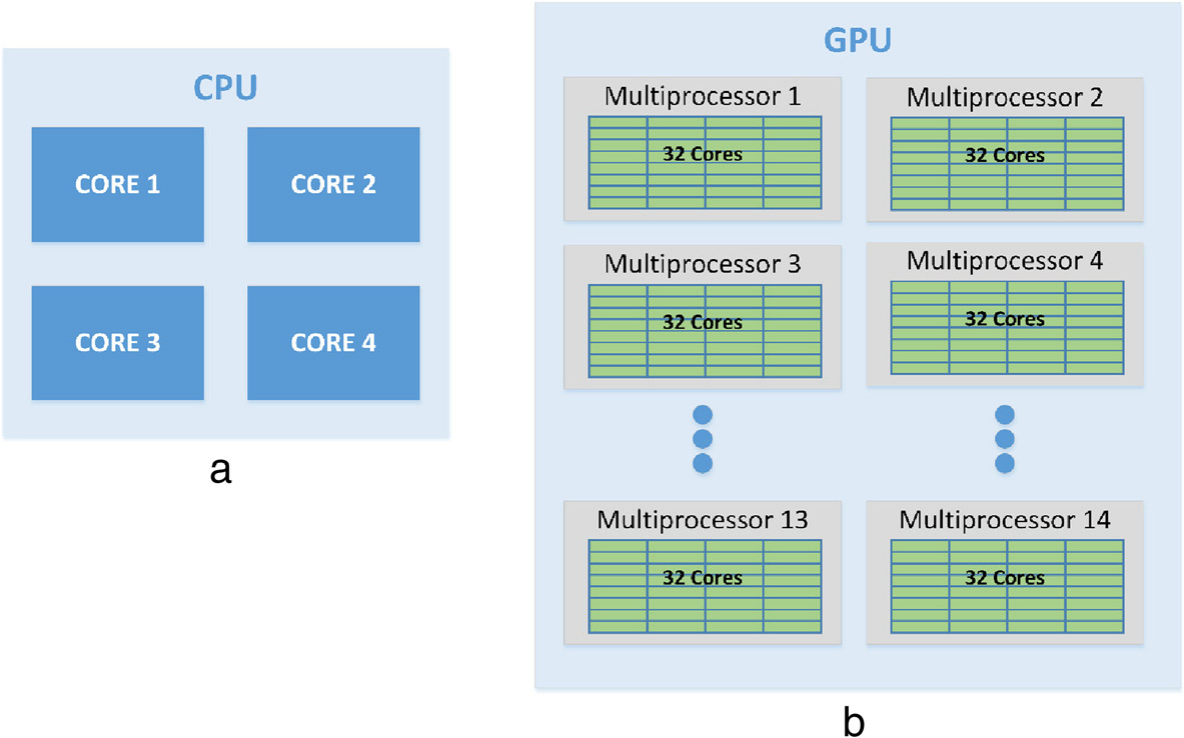
**A comparison between the architectures of CPU(a) and GPU(b) showing the arrangement of processing cores.**

Figure 3(a) shows a high-level CPU architecture with four cores. The parallelism in CPUs is mainly achieved through multithreading [29]. All the cores share the same memory. The task has to be broken down into threads. Each thread is executed in parallel on different CPU cores. Most of the programming languages provide libraries to create threads and use CPU parallelism. The most popular choice of such programming languages is Java. Since multicore CPUs have been around for several years, a large number of software applications and programming environments are well developed for this platform. The developments in CPUs are not at the same pace compared to GPUs. The number of cores per CPU is still in double digits with the processing power close to 10Gflops while a single GPU has more than 2500 processing cores with 1000Tflops of processing power. This massive parallelism in GPU makes it a more appealing option for parallel computing applications.

The drawback of CPUs is their limited number of processing cores and their primary dependence on the system memory for data access. System memory is limited to a few hundred gigabytes and this limits the size of the data that a CPU can process efficiently. Once the data size exceeds the system memory, disk access becomes a huge bottleneck. Even if the data fits into the system memory, CPU can process data at a much faster rate than the memory access speed which makes memory access a bottleneck. GPU avoids this by making use of DDR5 memory compared to a slower DDR3 memory used in a system. Also, GPU has high speed cache for each multiprocessor which speeds up the data access.

### Graphics processing unit (GPU)

Graphics Processing Unit (GPUs) is a specialized hardware designed to accelerate the creation of images in a frame buffer intended for display output [30]. Until the past few years, GPUs were primarily used for graphical operations such as video and image editing, accelerating graphics-related processing etc. However, due to their massively parallel architecture, recent developments in GPU hardware and related programming frameworks have given rise to GPGPU (general-purpose computing on graphics processing units) [31]. GPU has large number of processing cores (typically around 2500+ to date) as compared to a multicore CPU. In addition to the processing cores, GPU has its own high throughput DDR5 memory which is many times faster than a typical DDR3 memory. GPU performance has increased significantly in the past few years compared to that of CPU. Recently, Nvidia has launched Tesla series of GPUs which are specifically designed for high performance computing. Nvidia has released the CUDA framework which made GPU programming accessible to all programmers without delving into the hardware details. These developments suggest that GPGPU is indeed gaining more popularity. Figure 3(b) shows a high-level GPU architecture with 14 multiprocessors and 32 streaming processors per block. It usually has two levels of parallelism. At the first level, there are several multiprocessors (MPs) and within each multiprocessor there are several streaming processors (SPs). To use this setup, GPU program is broken down into threads which execute on SPs and these threads are grouped together to form thread blocks which run on a multiprocessor. Each thread within a block can communicate with each other and synchronize with other threads in the same block. Each of these threads has access to small but extremely fast shared cache memory and larger global main memory. Threads in one block cannot communicate with the threads in the other block as they may be scheduled at different times. This architecture implies that for any job to be run on GPU, it has to be broken into blocks of computation that can run independently without communicating with each other [32]. These blocks will have to be further broken down into smaller tasks that execute on an individual thread that may communicate with other threads in the same block.

GPUs have been used in the development of faster machine learning algorithms. Some libraries such as GPUMiner [33] implement few machine learning algorithms on GPU using the CUDA framework. Experiments have shown many folds speedup using the GPU compared to a multicore CPU.

GPU has its own drawbacks. The primary drawback is the limited memory that it contains. With a maximum of 12GB memory per GPU (as of current generation), it is not suitable to handle terabyte scale data. Once the data size is more than the size of the GPU memory, the performance decreases significantly as the disk access becomes the primary bottleneck. Another drawback is the limited amount of software and algorithms that are available for GPUs. Because of the way in which the task breakdown is required for GPUs, not many existing analytical algorithms are easily portable to GPUs.

### Field programmable gate arrays (FPGA)

FPGAs are highly specialized hardware units which are custom-built for specific applications [34]. FPGAs can be highly optimized for speed and can be orders of magnitude faster compared to other platforms for certain applications. They are programmed using Hardware descriptive language (HDL) [35]. Due to customized hardware, the development cost is typically much higher compared to other platforms. On the software side, coding has to be done in HDL with a low-level knowledge of the hardware which increases the algorithm development cost. User has to carefully investigate the suitability of a particular application for FPGA as they are effective only for a certain set of applications.

FPGAs are used in a variety of real-world applications [36, 37]. One example where FPGA was successfully deployed is in the network security applications [38]. In one such application, FPGA is used as a hardware firewall and is much faster than the software firewalls in scanning large amounts of network data [39]. In the recent years, the speed of multicore processors is reaching closer to that of FPGAs.

## Comparison of different platforms

We will now provide a more detailed comparison of different platforms using the (star) ratings, where 5 stars correspond to the best possible rating and 1 star corresponds to the lowest possible rating for any given platform for a particular characteristic. Table 2 compares the different platforms based on the following characteristics: scalability, data I/O performance, fault tolerance, real-time processing, data size support and the support for iterative tasks. Clearly, the first three characteristics are system/platform dependent and last three are application/algorithm dependent. We will provide more details about each of these characteristics and evaluate the “goodness” of each platform for that particular characteristic by providing the star ratings for each of the platform with respect to these characteristics. It should be noted that this table provides a mere qualitative comparisons between the platforms and is not intended to bring any quantitative judgments about these platforms. In other words, a rating of two stars for a platform should be interpreted as better than having a one star and does not necessarily mean that it is two times better than the platform with a one star. Similarly, if a platform X is rated with four stars, platform Y is rated with two stars, and platform Z is rated with one star, then it should be interpreted as platform X is significantly better than platform Z compared to platform Y. It should also be noted that it is almost impossible to quantify this significance in a general scenario and one can only do so in the context of a specific application or a problem at hand. However, we anticipate that this rating table provides a snapshot of the general strengths and weaknesses of various platforms available in the context of the critical characteristics related to big data analytics.

**Table 2:** **Comparison of different platforms (along with their communication mechanisms) based on various characteristics**

**Scaling type**	**Platforms (Communication Scheme)**	**System/Platform**			**Application/Algorithm**		
		**Scalability**	**Data I/O performance**	**Fault tolerance**	**Real-time processing**	**Data size supported**	**Iterative task support**
Horizontal scaling	Peer-to-Peer (TCP/IP)	★★★★★	★	★	★	★★★★★	★★
	Virtual clusters (MapRedce/MPI)	★★★★★	★★	★★★★★	★★	★★★★	★★
	Virtual clusters (Spark)	★★★★★	★★★	★★★★★	★★	★★★★	★★★
Vertical scaling	HPC clusters (MPI/Mapreduce)	★★★	★★★★	★★★★	★★★	★★★★	★★★★
	Multicore (Multithreading)	★★	★★★★	★★★★	★★★	★★	★★★★
	GPU (CUDA)	★★	★★★★★	★★★★	★★★★★	★★	★★★★
	FPGA (HDL)	★	★★★★★	★★★★	★★★★★	★★	★★★★

### Scalability

Scalability is defined as the ability of the system to handle growing amount of work load in a capable manner or its ability to be enlarged to accommodate that growth. In our case, scalability is considered to be the ability to add more hardware (scale up or scale out) to improve the capacity and performance of a system.

In this category, the virtual clusters and peer-to-peer networks will get 5 stars since these systems are highly scalable. In these two platforms, it is relatively easy to add more machines and scale out these systems to any extent needed. The HPC clusters will receive a 3 star rating because it becomes difficult to scale up these systems after a certain extent. HPC clusters can have thousands of cores but once deployed, they are expensive to scale up. 2 Stars for GPU shows that the scalability is not the strength of GPUs. There is a limit on the number of GPUs a single machine can have and adding more machines will create data transfer bottleneck over the network. 1 star rating for FPGA is due to the fact that once FPGA is developed and deployed, scaling up and modifying becomes extremely costly.

### Data I/O performance

Data I/O performance refers to the rate at which the data is transferred to/from a peripheral device. In the context of big data analytics, this can be viewed as the rate at which the data is read and written to the memory (or disk) or the data transfer rate between the nodes in a cluster. GPU and FPGA receive 5 stars since they have high throughput memory and the data I/O operations are extremely fast. The current generation GPUs are available with DDR5 memory which is many times faster than the DDR3 system memory. HPC clusters and Multicore will fall next in this category with 4 stars. These systems usually make use of system memory which is reasonably faster compared to disk access. Since HPC clusters and Multicore are usually single machines, network access is not a bottleneck.

Virtual clusters using the Spark framework receive 3 stars since Spark makes use of system memory which gives it an edge over Hadoop. Although system memory is fast, the data transfer between different nodes still takes place over the network. This makes the network access a bottleneck for data I/O. Virtual clusters using MapReduce over Hadoop receive 2 stars as they primarily read data from the disk which is a rather slow and time consuming process. In addition, the communication over the network degrades the performance.

Peer-to-peer systems are the worst in this category and will receive only 1 star. These systems use disks for the data access. In addition, the unmanaged and complex network scheme makes it very inefficient to aggregate the results over a single node and makes network communication even slower compared to the virtual clusters. These flaws degrade the data I/O performance.

### Fault tolerance

Fault tolerance [40] is the characteristic of a system to continue operating properly in the event of a failure of one or more components. Since we created this table with an intent to compare the platforms of similar capacity, we additionally consider the chances of failure in a system and give a high rating if system failures are extremely rare even though it may not have any fault tolerance mechanism. This enables us to make an unbiased comparison between unreliable systems with fault tolerance and reliable hardware with not so good fault tolerance mechanism.

Virtual clusters get 5 stars since they primarily use MapReduce or Spark, running on frameworks such as Hadoop which have efficient in-built fault tolerant mechanisms.

On the other hand, HPC clusters, Multicore, GPU and FPGA get 4 stars. Although none of these contain the state-of-the-art fault tolerant mechanisms, they have the most reliable and well-built hardware which makes the hardware failure an extremely rare event. They get a slightly lower rating compared to the virtual clusters because of the occasional hardware failures that can still happen in spite of their rarity. 1 star rating is given to peer-to-peer networks, which shows their inability to handle system failures. They do not have any in-built fault tolerance mechanisms and additionally these networks mainly consist of commodity machines which are highly susceptible to hardware failures.

### Real-time processing

Real-time processing of a system is its ability to process the data and produce the results strictly within certain time constrains. Real-time responses are often delivered in the order of milliseconds and sometimes microseconds depending on the application and the user requirements.

In this category, FPGA and GPU score 5 stars and outperform other platforms. GPU with its thousands of processing cores and high memory bandwidth is well suited for real-time data processing. Although their memory is limited, GPUs are optimized for speed and are often used for online processing. Similarly, FPGAs are specially built hardware optimized for speed and are suitable for real-time data processing.

Multicores and HPC clusters get 3 stars. They have reasonable real-time processing capabilities (though not as good as GPU and FPGA) with many processing cores and high bandwidth memory. Virtual clusters with 2 stars are not typically used for handling real-time processing tasks. They are slow in data I/O between the nodes and do not contain powerful and optimized hardware. Peer-to-Peer networks are the worst performers in this category and hence they receive only 1 star. They are slow with respect to real-time data processing because of the network communication overhead and commodity hardware.

### Data size supported

Data size support is the size of the dataset that a system can process and handle efficiently. In this category, peer-to-peer networks will receive 5 stars since they can handle even petabytes of data and can theoretically scale out to unlimited number of nodes. Virtual clusters and HPC clusters can handle terabytes of data and thus will receive 4 stars. Virtual clusters can scale up to tens of thousands of nodes and frameworks like Hadoop and Spark are capable of processing and handling such large datasets.

Multicore, GPU and FPGA are not well suited for processing large data sets. All these systems get 2 stars for the limited size of the data that they can support. GPUs have a limited on-board memory in the order of several gigabytes. Similarly, Multicore systems rely on system memory which can only be up to hundreds of gigabytes.

### Iterative tasks support

This is the ability of a system to efficiently support iterative tasks. Since many of the data analysis tasks and algorithms are iterative in nature, it is an important metric to compare different platforms, especially in the context of big data analytics.

HPC clusters, Multicore, GPU and FPGA score 4 stars in this category and all of them are highly suitable for iterative algorithms. However, all the iterative algorithms cannot be easily modified to run on each of these platforms which is the primary reason for giving 4 stars instead of 5 stars. These platforms are perfectly suitable for iterative algorithms since the result of one iteration can be easily used in the next iteration and all the parameters can be stored locally. Processes can reside and can keep running as long as the machine is running [41].

Virtual clusters using spark receive only 3 stars. The ability of the spark framework to use system memory for storing the data significantly reduces the data I/O overhead. The iterative tasks can still run in such cases but not as efficiently as the other platforms mentioned earlier. Data still has to be written to the memory after every iteration.

Virtual clusters using MapReduce and peer-to-peer frameworks are not designed to handle iterative tasks and hence received only 2 stars. MapReduce is not designed for iterative processing and the data has to be written onto the disk after every iteration, thus making the disk I/O a huge bottleneck. Some recent developments such as HaLoop improves MapReduce performance for iterative tasks to a certain extent which is why it was not given a 1 star. Peer-to-peer networks using TCP/IP are overwhelmed by the network communication overhead. To combine the results from different nodes after every iteration presents a significant challenge due to the complex network structure.

## How to choose a platform for big data analytics?

The star ratings provided in Table 2 gives a bird’s eye view of the capabilities and features of different platforms. The decision to choose a particular platform for a certain application usually depends on the following important factors: data size, speed or throughput optimization and model development. We will now provide more details about each of these factors.

### Data size

The size of data that is being considered for processing is probably the most important factor. If the data can fit into the system memory, then clusters are usually not required and the entire data can be processed on a single machine. The platforms such as GPU, Multicore CPUs etc. can be used to speed up the data processing in this case. If the data does not fit into the system memory, then one has to look at other cluster options such as Hadoop, Spark etc. Again, Hadoop and Spark clusters can handle large amount of data but Hadoop has well developed tools and frameworks although it is slower for iterative tasks. The user has to decide if he needs to use off-the-shelf tools which are available for Hadoop or if he wants to optimize the cluster performance in which case Spark is more appropriate.

### Speed or throughput optimization

Here, speed refers to the ability of the platform to process data in real-time whereas throughput refers to the amount of data that system is capable of handling and processing simultaneously. The users will need to be clear about whether the goal is to optimize the system for speed or throughput. If one needs to process large amount of data and do not have strict constraints on the processing time, then one can look into systems which can scale out to process huge amounts of data such as Hadoop, Peer-to-Peer networks, etc. These platforms can handle large-scale data but usually take more time to deliver the results. On the other hand, if one needs to optimize the system for speed rather than the size of the data, then they need to consider systems which are more capable of real-time processing such as GPU, FPGA etc. These platforms are capable of processing the data in real-time but the size of the data supported is rather limited.

### Training/Applying a model

In data analytics, training of the model is typically done offline and it usually takes a significant amount of time. A model is typically applied in an online environment where the user expects the results within a short period of time (almost instantaneously). This creates a strong need for investigating different platforms for training and applying a model depending on the end-user application. Usually, during the training process, the user needs to deal with large amount of training data and since training is done offline, the processing time is not critical. This makes horizontal scale out platforms suitable for training a model. When a model is applied, results are expected in real-time and vertical scale up platforms are usually preferred. GPUs are preferable over other platforms when the user has strict real-time constraints and the data can be fit into the system memory. On the other hand, the HPC clusters can handle more data compared to GPUs but are not suitable for real-time processing compared to GPUs.

### Practical implications

This work can provide useful insights for a wide variety of practical applications. Many users and researchers working in different areas of big data ranging from real-time systems to large-scale data processing applications can assess the advantages and disadvantages of the platforms they are currently using. This survey will be immensely helpful for them to understand if they can improve the existing systems by choosing the right platform. The strengths and weaknesses of each of the platforms are clearly highlighted in this paper. More specifically, for big data applications, there is often a trade-off between the real-time analysis requirements and the scalability of the data being processed. For the former, GPUs will be optimal choice and for the latter horizontal scaling platforms such as Hadoop and Spark are optimal choices. For example, in some of the recommendation problems dealing with millions of users, it is extremely important to have a scalable platform that can handle huge amounts of data processing and it is not absolutely required to have the results in a real-time manner. In web search applications, due to the billions of webpages available, the webpage indexing process will require a highly scalable platform which can accurately index the webpages offline. However, for the actual online component where the user enters the query and the results are expected to be obtained in real-time, vertical scaling platforms might be more suitable. Hence, depending on the application, one can probably define the most critical needs and then choose the right kind of platform accordingly. In the previous section, we characterized several such needs for practical applications.

## K-means clustering on different platforms

In order to provide more insights into the analytics algorithms on different platforms, we will demonstrate the implementation of the K-Means clustering algorithm on these platforms presented so far. The choice of the K-Means algorithm was made not only because of its popularity and wide usage [42, 43], but also due to some of its critical elements that can demonstrate the ability of various platforms in handling other analytics procedures. Some of these characteristics include:

- Iterative nature of the algorithm wherein the current iteration results are needed before proceeding to the next iteration.
- Compute-intensive task of calculating the centroids from a set of datapoints.
- Aggregation of the local results to obtain a global solution when the algorithm is parallelized.

**Figure 4:**
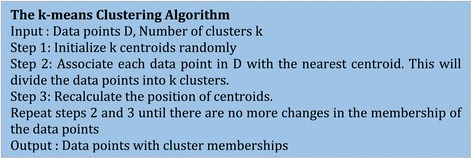
**The pseudocode of the K-means clustering algorithm.**

It should be noted that many of the analytics algorithms share atleast some of these characteristics. Hence, it is important to understand how these characteristics of K-means clustering algorithm are being handled using different platforms. Figure 4 explains the different steps involved in a basic K-means clustering algorithm. The algorithm starts by initializing the cluster centroids. In the next step, each data point is associated with closest centroid and in the third step, the centroids are recalculated for all the associated data instances for a given cluster. The second and third steps are repeated until the centroids converge (or after a pre-defined number of iterations).

We will now discuss the implementation details of this algorithm on different platforms to get a deeper understanding of how such iterative algorithms are modified to fit different communication schemes.

### K-means on MapReduce

**Figure 5:**
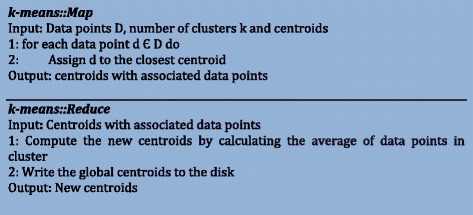
**Pseudocode of MapReduce based K-means clustering algorithm.** The first part shows the map function and the second part shows the reduce function.

MapReduce is not an ideal choice for iterative algorithms such as K-Means clustering. This will be clearly shown in this section as we explain the K-Means clustering using MapReduce. The pseudocode for mapper and reducer functions for k-means clustering algorithm is given in Figure 5. Basically, mappers read the data and the centroids from the disk. These mappers then assign data instances to clusters. Once every mapper has completed their operation, reducers compute the new centroids by calculating the average of data points present in each cluster. Now, these new centroids are written to the disk. These centroids are then read by the mappers for the next iteration and the entire process is repeated until the algorithm converges. This shows the disk access bottleneck of MapReduce for iterative tasks as the data has to be written to the disk after every iteration.

### K-means on MPI

**Figure 6:**
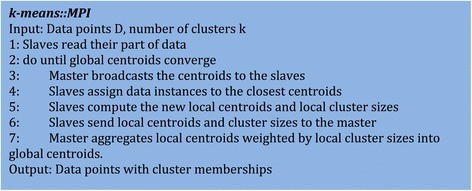
**Pseudocode of k-means clustering algorithm using MPI in a master–slave configuration.**

MPI [44] typically have a master–slave setting and the data is usually distributed among the slaves. Figure 6 explains the pseudocode for K-means using MPI. In the first step, the slaves read their portion of the data. In the second step, the master broadcasts the centroids to the slaves. Next, the slaves assign data instances to the clusters and compute new local centroids which are then sent back to the master. Master will then compute new global centroids by aggregating local centroids weighted by local cluster sizes. These new global centroids are then again broadcasted back to the slaves for the next iteration of K-means. In this manner, the process continues until the centroids converge. In this implementation, the data is not written to the disk but the primary bottleneck lies in the communication when MPI is used with peer-to-peer networks since aggregation is costly and the network performance will be low.

### K-means on GPU

**Figure 7:**
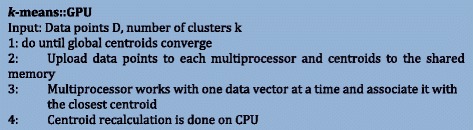
**The pseudocode of the K-means clustering algorithm on GPU.**

GPU has a large number of processing cores. Hence, in order to effectively utilize all the cores, the algorithm will need to be modified cautiously. Figure 7 shows the pseudocode of K-means using GPU. In the case of K-means, each processor is given a small task (assigning a data vector to a centroid). Also, a single core in a GPU is not very powerful which is why the centroid recalculation is done on the CPU. The centroids are uploaded to the shared memory of the GPU and the datapoints are partitioned and uploaded into each multiprocessor. These multiprocessors work on one data vector at a time and associate it with the closest centroid. Once all the points are assigned to the centroids, CPU recalculates the centroids and again will upload the new centroids to the multiprocessors. This process is repeated until the centroids converge or until a pre-defined number of iterations are completed. Another aspect to consider here is the density of the data. If the data is sparse, many multiprocessors will stall due to scarcity of data vectors to compute, which will eventually degrade the performance. In a nutshell, the performance of GPUs will be the best when the data is relatively denser and when the algorithm is carefully modified to take advantage of processing cores.

### K-means on other platforms

K-means implementation on Spark is similar to the MapReduce-based implementation described in Section K-means on MapReduce. Instead of writing the global centroids to the disk, they are written into the memory which speeds up the processing and reduces the disk I/O overhead. In addition, the data will be loaded into the system memory in order to provide faster access. The K-means clustering on CPU involves multithreading where each thread associates a data vector to a centroid and finally the centroids are recomputed for the next iteration. On the other hand, K-means implementation on FPGA depends upon the FPGA architecture used and may differ significantly depending on the type of FPGA being used.

## Conclusion and future directions

This paper surveys various data processing platforms that are currently available and discusses the advantages and drawbacks for each of them. Several details on each of these hardware platforms along with some of the popular software frameworks such as Hadoop and Spark are also provided. A thorough comparison between different platforms based on some of the important characteristics (such as scalability and real-time processing) has also been made through star based ratings. The widely used k-means clustering algorithm was chosen as a case study to demonstrate the strengths and weaknesses of different platforms. Some of the important characteristics of k-means algorithm such as its iterative nature, compute-intensive calculations and aggregating local results in a parallel setting makes it an ideal choice to better understand the various big data platforms. It should be noted that many of the analytical algorithms share these characteristics as well. This article provides the readers with a comprehensive review of different platforms which can potentially aid them in making the right decisions in choosing the platforms based on their data/computational requirements.

The future work involves investigating more algorithms such as decision trees, nearest neighbor, pagerank etc. over different platforms. For empirical evaluation, different experiments involving varying data size and response times can be performed over various platforms for different algorithms. Through such an analysis we will get valuable insights which can be useful in many practical and research applications. One other important direction of research will be to choose the right platform for a particular application. Based on the specific application needs, one can tailor their platform specific factors such as the amount of hard disk, memory and the speed required for optimally running the application. This study will provide a first step to analyze the effectiveness of each of the platforms and especially the strengths of them for handling real-world applications. Another direction will be to investigate the possibility of combining multiple platforms to solve a particular application problem. For example, attempting to merge the horizontal scaling platforms such as Hadoop with vertical scaling platforms such as GPUs is also gaining some recent attention. This involves several non-trivial tasks not only at the platform level but also becomes challenging to decompose the algorithm into parts and running various algorithmic components in various platforms. A combination of platforms might be more suitable for a particular algorithm and can potentially resolve the issue of making it highly scalable (through horizontal scaling) as well as performing real-time analysis (through vertical scaling).
